# Acne vaccine targeting *Cutibacterium acnes* secretory lipase suppresses the bacteria-induced pro-inflammatory interleukin-6 and macrophage inflammatory protein-2 production

**DOI:** 10.5114/bta/221530

**Published:** 2026-06-27

**Authors:** Indira Putri Negari, Azka Narari Khoerunnisa, Chun Ming Huang, Shinta Marito, Uuganbayar Gankhuyag, Mai Trinh Tang Nguyen, Aswin Rafif Khairullah, Cindy Mutiara Septani

**Affiliations:** 1 Research Center for Vaccine and Drugs, National Research and Innovation Agency (BRIN), Bogor, West Java, Indonesia; 2 Department of Biology, Faculty of Military Mathematics and Natural Sciences, Universitas Pertahanan, Bogor, West Java, Indonesia; 3 Department of Biomedical Sciences and Engineering, National Central University, Zhongli, Taoyuan, Taiwan; 4 Department of Pharmacy, Universitas Katolik Widya Mandala Surabaya, Surabaya, East Java, Indonesia; 5 Department of Fundamental of Science, School of Nursing, Mongolian National University of Medical Sciences, Ulaanbaatar, Mongolia; 6 Department of Life Sciences, National Central University, Zhongli, Taoyuan, Taiwan; 7 Immunology Research Center, National Health Research Institutes, Zhunan, Miaoli, Taiwan; 8 Research Center for Veterinary Science, National Research and Innovation Agency (BRIN), Bogor, West Java, Indonesia; 9 Research Center of Polymer Technology, National Research and Innovation Agency (BRIN), South Tangerang, Banten, Indonesia

**Keywords:** *Cutibacterium acnes*, IL-6, lipase, MIP-2, palmitic acid

## Abstract

**Background:**

Limited attention paid to acne vulgaris has hindered progress in studying its pathological mechanisms and developing new treatments. While direct evidence confirming *Cutibacterium acnes* (*C. acnes*) as the initial trigger of acne vulgaris is scarce, the bacterium clearly drives inflammation within acne lesions.

**Materials and methods:**

Mass spectrometric analysis demonstrated that *C. acnes* lipase digested triglycerides into various fatty acids, which increased sebum production in sebocytes. The secretory lipase was selected as an immunogenic antigen to generate antibodies in mice. Serum from mice vaccinated with *C. acnes* lipase was used to assess antibody titers via test strips. Pro-inflammatory interleukin-6 (IL-6) and macrophage inflammatory protein-2 (MIP-2) expression were measured by enzyme-linked immunosorbent assay. Passive immunization with neutralization antibodies to lipase was conducted by administering anti-lipase serum co-cultured with *C. acnes*.

**Results:**

Palmitic acid, a lipase-derived fatty acid, was identified as a major pro-inflammatory stimulus in sebocytes, confirming the central role of lipase in acne inflammation. Mice vaccinated with *C. acnes* lipase showed protection against *C. acnes*-induced inflammation in an ear model. Therefore, neutralization antibodies to lipase significantly reduced *C. acnes*-induced production of pro-inflammatory IL-6 and MIP-2.

**Conclusions:**

The *C. acnes* secretory lipase is a key virulence factor contributing to acne inflammation. Vaccination with *C. acnes* lipase significantly reduced bacterial colonization and the production of pro-inflammatory IL-6 and MIP-2. These findings support the potential of *C. acnes* lipase-targeted vaccines as a novel, antigen-specific strategy for both the treatment and prevention of acne vulgaris.

## Introduction

*Cutibacterium acnes* (*C. acnes*) is an anaerobic, Gram-positive bacterium that resides within pilosebaceous follicles as part of the normal skin microbiota. However, overproliferation and colonization within these hair follicles strongly implicate *C. acnes* in triggering inflammatory acne vulgaris. This condition is the most prevalent skin disorder, affecting up to 85% of individuals at some point in their lifetime (Dréno et al. 2018; Kim et al. 2020; Hazarika 2021; Singh et al. 2022; Ahle et al. 2023). Acne vulgaris is recognized as the eighth most common dermatological condition worldwide, with the Global Burden of Disease Study estimating its overall prevalence at 9.38% across all age groups. Its prevalence rates vary by country and age; adolescent acne rates range from 35% to nearly 100%, while the prevalence among adults is 0.74% in clinical populations (Heng and Chew 2020; Shah et al. 2021).

Treatment options for acne vulgaris include topical and systemic retinoid therapies, such as isotretinoin, benzoyl peroxide, azelaic acid, and topical antibiotics (Vasam et al. 2023). Topical or combination therapies are standard for mild-to-moderate acne, while systemic treatments are reserved for severe nodular or scarring forms (Fox et al. 2016; Sevimli Dikicier 2019; Greydanus et al. 2021). However, these medications often cause side effects, such as skin irritation, and frequently require prolonged use (Dessinioti and Katsambas 2017; Dréno et al. 2020; Landis 2020; Biazzo et al. 2025). In addition, *C. acnes* secretes enzymes, including lipases, proteases, and hyaluronidases, which degrade sebum and extracellular matrix components, thereby damaging tissue and amplifying inflammation (Singh et al. 2022; Fan et al. 2024; Wu et al. 2025). This process is further mediated by the release of pro-inflammatory cytokines, such as interleukin-6 (IL-6) (Zhou et al. 2013), and chemokines like macrophage inflammatory protein-2 (MIP-2), which promote immune cell recruitment and activation (Choi et al. 2019). Persistent inflammation not only drives lesion development but also causes tissue remodeling and scarring, underscoring its central role in acne pathogenesis (Dréno et al. 2015; Cavallo et al. 2022; Firlej et al. 2022).

Owing to its critical role in initiating and sustaining inflammation within acne lesions, *C. acnes* is now a primary therapeutic target (Wang et al. 2018; Mayslich et al. 2021; Cavallo et al. 2022; Chen et al. 2025). Therefore, vaccines targeting *C. acnes*-induced pathogenesis may offer significant clinical value to prevent and treat acne vulgaris (Nakatsuji et al. 2008; Simonart 2013; Liu et al. 2015; Wang et al. 2018; Keshari et al. 2019; Burkhart 2024). The presence of immunoglobulin G (IgG)-coated bacteria within the comedones of acne patients (Burkhart 2024) indicates that antibodies produced through immunization can penetrate comedonal structures and interact with *C. acnes*. Moreover, therapeutic antibodies that target inflammation induced by secreted virulence factors, rather than bacterial cells, may preserve commensal *C. acnes*, minimizing disruption to the skin microbiota (Liu et al. 2015). Along with other free fatty acids and virulence factors, lipase generated by *C. acnes* drives bacterial pathogenicity (Borrel et al. 2019; Kim et al. 2020; Mayslich et al. 2021). In particular, free fatty acids like oleic acid and palmitic acid are closely linked to acne severity (Lovászi et al. 2017; Tabri 2019; Choi et al. 2019; Nakase et al. 2022; Kovács et al. 2023).

In this study, we investigated whether *C. acnes* secretory lipase functions as a key virulence factor contributing to acne vulgaris inflammation in a mouse model. Specifically, we evaluated whether vaccination with *C. acnes* lipase significantly decreases bacterial colonization and the production of pro-inflammatory IL-6 and MIP-2. Consequently, this research characterizes the immunogenicity and therapeutic potential of *C. acnes* lipase.

## Materials and methods

### Animals

The Institute of Cancer Research (ICR) and C57BL/6 IL-6 knockout mice (8–9 weeks old, female) were euthanized using 10% CO_2_ in a sealed chamber (Boivin 2017; Russell et al. 2021). Mice were housed under controlled environmental conditions at an ambient temperature of 23 ± 2°C in standard cages with wood-chip bedding and *ad libitum* access to food and water (Russell et al. 2021). Animals were allowed to acclimate to the housing conditions for 3–7 days before data collection. Each experimental group comprised three randomly selected mice (*n* = 3 per group), and blinding was applied.

### Bacterial culture

A single colony of *C. acnes* bacteria was inoculated into Reinforced Clostridium Medium (RCM) (Oxford, Hampshire, England) and cultured at 37°C under anaerobic conditions until the logarithmic growth phase. Bacterial pellets were collected by centrifugation at 5,000 × g for 10 min, washed, and resuspended in phosphate-buffered saline (PBS).

### Cell culture

Sebocytes were cultured in Sebomed Basal Medium (Biochrom, Berlin, Germany) supplemented with 5 ng/ml human recombinant epidermal growth factor (Sigma, St. Louis, MO, USA) and 10% volume/volume (v/v) heatinactivated fetal bovine serum, and then incubated at 37°C in 5% (v/v) CO_2_. Cells were cultured to 90% confluence before subculturing. Finally, the cells were seeded at a density of 8 × 10^5^ cells/ml in 10-cm Petri dishes containing 10 ml of media, which was renewed once every 2 days.

### Expression of pro-inflammatory IL-6 in sebocytes treated with fatty acid

Sebocytes were cultured in 96-well culture plates and treated with 10 μM palmitoleic acid and 10 μM palmitic acid for 24 h. Samples treated with PBS served as controls. The expression of pro-inflammatory IL-6 in the cell culture medium was measured using enzymelinked immunosorbent assay (ELISA).

### Preparation of lipidome metabolome extract using chloroform and methanol

Sebocytes (1 × 10^7^) were treated with 50 μg of recombinant green fluorescent protein (GFP) as a control, or 50 μg of lipase dissolved in 100 μl PBS. Cells were harvested, combined with 1 ml of water and 3.75 ml of a chloroform/methanol mixture (1 : 1, v/v), and then vortexed for 10–15 min. Subsequently, 1.25 ml of chloroform was added, followed by vortexing for 1 min, and then 1.25 ml of water was added and the mixture was vortexed again for 1 min. The sample was centrifuged at 13,000 × rpm for 10 min. The protein disc was carefully pierced with a pipette tip, and the lower organic phase was collected and transferred to a new tube. The organic solvent was evaporated under vacuum at room temperature (RT), and the dried sample was stored at –80°C. Before analysis, the lipid extract was reconstituted in 50 μl of a chloroform/methanol mixture (1 : 1, v/v).

### Gas chromatography–mass spectrometry analysis of sebocyte metabolism

Lipid samples were analyzed using an HP 5890 gas chromatograph coupled with an HP 5971A mass spectrometer, an HP 7673A autosampler, and an Agilent Rtx-5MS column (30 m × 0.25 mm × 0.25 μm). A non-polar lipid standard mixture was used for compound identification and calibration. Relative lipid quantities were determined by comparing the single-ion response ratios of each analyte with the nearest eluting internal standard using HP ChemStation software (Agilent Technologies, Santa Clara, CA, United States, USA) for peak normalization (Kao et al. 2021; Keshari et al. 2020). Internal standards compensate for variability during extraction, sample preparation, and instrument response and are widely recommended for gas chromatography–mass spectrometry (GC-MS) analysis (Capoun and Krykorkova 2020). Quality control was maintained using calibration curves generated from multiple concentration levels of the Free Fatty Acids Test Standard (Restek Corporation) and by monitoring chromatographic peak areas or selected ion responses to verify consistent analytical performance (Capoun and Krykorkova 2020; Kao et al. 2021; Keshari et al. 2020).

### Molecular cloning and expression of recombinant C. acnes lipase

Plasmids carrying the gene encoding either *C. acnes* lipase (accession number: YP_056770.1) or GFP (accession number: JS0692; control) were transformed into *Escherichia coli* (*E. coli*) BL21 (DE3) competent cells (Invitrogen, Carlsbad, CA, USA). Then, *E. coli* BL21 (DE3) clones expressing lipase or GFP were selected on Luria-Bertani (LB) (Biokar Diagnostics, Beauvais, France) medium containing 100 μg/ml ampicillin (Sigma-Aldrich, St. Louis, MO, USA). An aliquot of the overnight culture from each group was diluted with LB medium (1 : 10) and incubated at 37°C until it reached an OD_600_ of about 0.6–0.8. Isopropyl-β-D-thiogalactoside (final concentration of 1 mM; Sigma, St. Louis, MO, USA) was added to the culture and incubated for 4 h at 30°C to induce protein expression. The recombinant lipase or GFP with 6 × His tag was purified by a ProBond™ Purification System (Invitrogen Carlsbad, CA, USA), followed by centrifugation with 10,000 molecular weight cut-offs Spin-X^®^ UF 6 concentrator (Cityva, Little Chalfont, Buckinghamshire, UK). Subsequently, the purified recombinant lipase or GFP was run on a 20% sodium dodecyl sulfate–polyacrylamide gel and stained with Coomassie Brilliant Blue R-250 (Biorad, Hercules, CA, USA) to detect protein expression.

### Vaccination of mice and antibody detection

A 50 μg of recombinant GFP or lipase was dissolved in 100 μl PBS and mixed with an equal volume (100 μl) of 2% Alhydrogel (InvivoGen, San Diego, CA, USA). ICR mice were subcutaneously vaccinated on the dorsal skin three times at 2-week intervals. Mice were euthanized 2 weeks after the final vaccination to collect serum samples containing IgG, which were detected by a microplate reader at 450 nm subtracted from 570 nm (OD_570-450_). For quantification, serum IgG antibody response was measured by ELISA, by coating 0.1 μg/well of recombinant GFP or lipase in 100 μl PBS at RT overnight. Next, the plate was blocked with 2% skimmed milk in PBS at RT for 1 h, and then incubated with diluted serum from mice vaccinated with GFP or lipase for 1 h. Subsequently, horseradish peroxidase (HRP)-conjugated rabbit anti-mouse IgG (1 : 10,000, Promega, WI, USA) was added and incubated for 1 h. HRP activity was determined with NeA-Blue tetramethylbenzidine substrate (Clinical Science Product, Mansfield, MA, USA) for 1 h. Finally, the reaction was quenched by adding sulfuric acid as a stop solution (2 N; Merck, Billerica, MA, USA), and the optical density of each well was measured at 450 nm subtracted from 570 nm (OD_570-450_). For test strip fabrication, an immunochromatographic strip consisting of a sample pad, conjugate release pad, and absorbent pad was assembled with a nitrocellulose (NC) membrane (Lovászi et al. 2017), with gold nanoparticles incorporated into the assembly. The NC membrane was spotted with Bovine Serum Albumin, anti-lipase or *C. acnes* serum lysates, and rabbit anti-mouse IgG secondary antibody at the designated (test–lower, control–upper) lines. The sample pad was loaded by soaking in 2.5% (v/v) serum (200 μl).

### Immunogenicity of C. acnes lipase

ICR mice (*n* = 3 per group) were anesthetized using isoflurane (Panion and BF Biotech Inc., Taiwan). The ears of the ICR mice were intradermally injected with either recombinant lipase or GFP (5 μg in 10 μl PBS). Then, ear tissue homogenates were quantified by ELISA to evaluate the expression of pro-inflammatory IL-6.

### Immune protection of anti-lipase/C. acnes in mice

Live *C. acnes* (10^7^ colony-forming units [CFU] in 10 μl of media) incubated with anti-lipase or GFP was subcutaneously injected into the central region of the right ear of the mice, while PBS as a control was injected in the left ear. After 72 h, ear tissue homogenates were collected and evaluated for the expression of proinflammatory IL-6 by ELISA.

### Neutralization assays

*C. acnes* (10^7^ CFU) was pre-incubated with 5% (v/v) of anti-lipase or anti-GFP serum in culture medium at 37°C for 1 h. Complement in the serum was heat-inactivated at 58°C for 30 min. For in vivo neutralization assays, *C. acnes* (10^7^ CFU) pre-treated with anti-lipase or anti-GFP serum was intradermally injected into the ears of ICR mice and incubated for 72 h. For *in vitro* neutralization assays, pre-treated *C. acnes* (10^7^ CFU) was added to sebocyte cultures (8 × 10^5^) and incubated for 12 h (Nakatsuji et al. 2008).

### Bacterial loads in the mouse ears

Following the intradermal injection of *C. acnes* along with either anti-lipase or GFP serum, the thickness of mouse ears was measured at 24, 48, and 72 h post-injection using an electronic digital caliper (Mitutoyo, Kanagawa, Japan). Redness or erythema of the ears was photographed using a USB digital microscope (GM019-#1, Global Camera Manufacturer Corp, Taipei, Taiwan). Ear tissues were then homogenized in 200 μl of sterile PBS using a tissue grinder. Serial dilutions (1 : 10^0^–1 : 10^5^) of the homogenates were plated on RCM agar to determine the CFU of *C. acnes*. Plates were incubated at 37°C for 3 days under anaerobic conditions using a Gas-Pak system. Colony formation was observed only in the ears injected with *C. acnes*, while no bacterial growth was detected in PBS-injected control ears.

### ELISA

Ear samples from ICR or IL-6 knockout mice were collected 72 h after the injection of GFP/lipase or anti-GFP/lipase serum. Then, ear tissues were excised and homogenized with T-PER™ Tissue Protein Extraction Reagent (Thermo Fisher, Waltham, MA, USA), supplemented with ethylenediaminetetraacetic acid-free protease inhibitor cocktail (Sigma-Aldrich, St. Louis, MO, USA). The ear homogenates were centrifuged at 15,000 rpm for 30 min at 4°C, and the supernatants were collected. To measure the concentrations of pro-inflammatory IL-6 and MIP-2 in the supernatants of ear homogenates and sebocytes, a sandwich ELISA was performed using a Quantikine mouse IL-6 and MIP-2 kit (R&D Systems, Minneapolis, MN, USA).

### Statistical analysis

Data are presented as the mean ± standard deviation from a minimum of three independent experiments. Statistical significance was evaluated using an unpaired *t*-test or a one-way analysis of variance followed by Tukey’s post hoc analysis, performed with SigmaStat software (Jandel Scientific, Palo Alto, CA, USA). *P*-values of < 0.05 (*), < 0.01 (**), and < 0.001 (***) were considered statistically significant. Different letters denote significant differences between groups (*p* < 0.05) (Negari et al. 2021).

## Results

### GC–MS analysis of fatty acids in sebocytes and cytokine release

**Figure 1:**
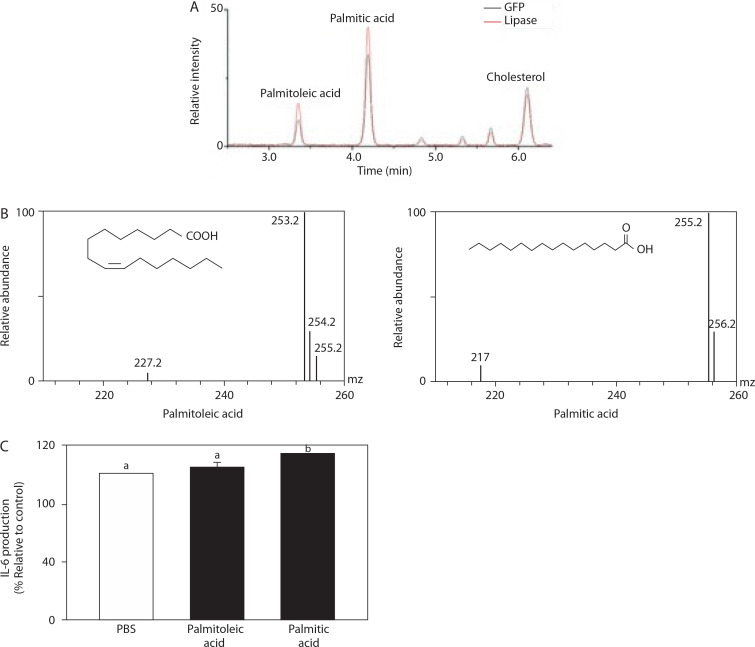
Gas chromatography–mass spectrometry analysis of fatty acids in sebocytes incubated with lipase or green fluorescent protein (GFP) for 24 h. (**A**) Mass spectra and retention times indicated palmitoleic acid, palmitic acid, and cholesterol. (**B**) The ion chromatogram and mass spectrum of palmitoleic and palmitic acids. (**C**) Sebocytes were treated with phosphate-buffered saline (PBS) as a control, 10 μM palmitoleic acid, or 10 μM palmitic acid for 24 h. The production of proinflammatory interleukin-6 (IL-6) was quantified by enzymelinked immunosorbent assay (ELISA). Different letters denote significant differences between groups (*p* < 0.05; *n* = 3)

To identify fatty acids in sebocytes, cells (1 × 10^7^) were treated with 50 μg of recombinant GFP or lipase dissolved in 100 μl PBS. For targeted metabolite analysis, all detected metabolites had been previously characterized using GC-MS standards, based on accurate mass and retention time. For each compound, the peak area was determined from the extracted ion chromatogram corresponding to its specific retention time. Twelve compounds were detected in both lipase and GFP groups (Supplementary Table 1), demonstrating that lipase hydrolyzes lipids in sebocytes into a variety of fatty acids. Notably, palmitoleic and palmitic acid concentrations were significantly increased in the lipase group (Figures 1A and 1B). Several studies have reported that lipase can break down lipids into palmitoleic and palmitic acids, which may be associated with acne severity (Zhou et al. 2013; Jung et al. 2021; de Souza Pereira 2023; Al-Saedi et al. 2022). Furthermore, we investigated the potential role of palmitoleic and palmitic acids in inducing inflammatory responses in sebocytes by measuring the production of pro-inflammatory IL-6. In the presence of palmitic acid, pro-inflammatory IL-6 expression was significantly upregulated compared to the PBS-treated control (Figure 1C). A 10 μM concentration was selected for both palmitic and palmitoleic acids, as previous studies have shown that this concentration is physiologically relevant and can induce measurable biological responses, including inflammatory signaling and lipid metabolism regulation, without causing significant cytotoxicity (Ishikawa et al. 2026; Murakami et al. 2020). Additionally, a 24-h incubation period was selected, as it is widely applied in in vitro studies to allow for sufficient cellular response and gene expression changes following fatty acid treatment (Chlebicz and Śliżewska 2020; Figueiredo et al. 2017; Hipp et al. 2017). Conversely, sebocytes treated with palmitoleic acid showed a less pronounced difference in pro-inflammatory IL-6 levels. This finding demonstrates that sebocytes respond specifically to palmitic acid stimulation by upregulating inflammatory enzyme expression and increasing the production and secretion of pro-inflammatory cytokines. This aligns with previous research indicating that palmitic acid elevates intracellular lipid accumulation and stimulates pro-inflammatory IL-6 secretion in sebocytes (Choi et al. 2019; Törőcsik et al. 2021).

### Antibody production in mice vaccinated with C. acnes lipase

**Figure 2:**
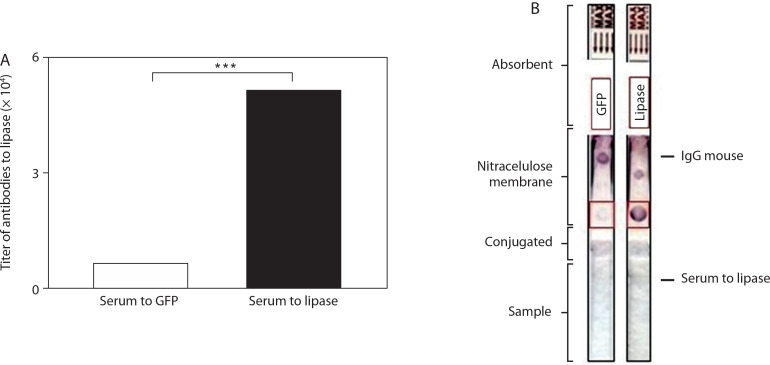
The production of antibodies to lipase. The Institute of Cancer Research mice were subcutaneously vaccinated with lipase or green fluorescent protein (GFP) and exhibited detectable antibodies immunoglobulin G (IgG) to lipase. (**A**) The titer of antibodies to lipase. (**B**) The production of antibodies to lipase or GFP and their cross-reactivity with mouse serum to lipase were detected in test strips with NC membranes spotted with lipase or GFP (lower position) and rabbit anti-mouse IgG secondary antibody (upper position). Data are expressed as mean ± standard deviation (*p < 0.05; **p < 0.01; ***p < 0.001 vs. control, *n* = 3)

**Figure 3:**
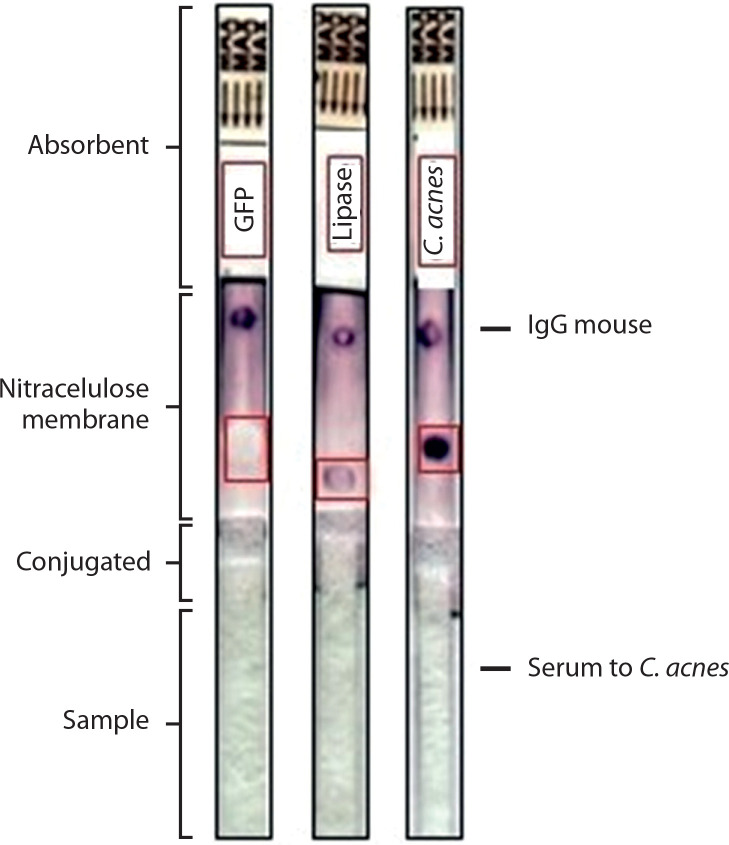
The production of antibodies to *Cutibacteriumacnes* (*C. acnes*). The Institute of Cancer Research mice were subcutaneously vaccinated with lipase, green fluorescent protein (GFP), or *C. acnes* and exhibited detectable antibodies immunoglobulin G (IgG) to lipase and *C. acnes*. The production of antibodies to lipase, GFP, or *C. acnes* and their cross-reactivity with mouse serum to *C. acnes* were detected in test strips with NC membranes spotted with lipase, GFP, or *C. acnes* (lower position) and rabbit anti-mouse IgG secondary antibody (upper position)

To examine antibody titers to lipase and validate the test strip fabrication, we vaccinated ICR mice with *E. coli* overexpressing lipase or GFP. Two weeks postvaccination, serum from lipase-immunized mice exhibited significantly higher IgG titers against lipase than serum from GFP-immunized controls, indicating that lipase exhibits immunogenic properties and can elicit a robust immune response. This result is supported by previous studies demonstrating that lipase influences immune activity in acne vulgaris (Ahle et al. 2023; Kovács et al. 2023). Serial dilution of the antibody titer to lipase (1 : 51,200) was performed to detect and quantify antibodies (Figure 2A). Moreover, immunochromatographic test strips were developed by coating with GFP, lipase, or lysates of *C. acnes*. Serum collected from mice vaccinated with lipase (Figure 2B) or lysates of *C. acnes* (Figure 3) was applied to the test strips. As shown in Figures 2B, and 3, lipase or lysates of *C. acnes*, but not GFP, were recognized by antibodies in both anti-lipase serum and *C. acnes*, suggesting that vaccination with lipase provoked antibodies that can cross-react with *C. acnes*.

### Immunogenicity of C. acnes lipase in mice

**Figure 4:**
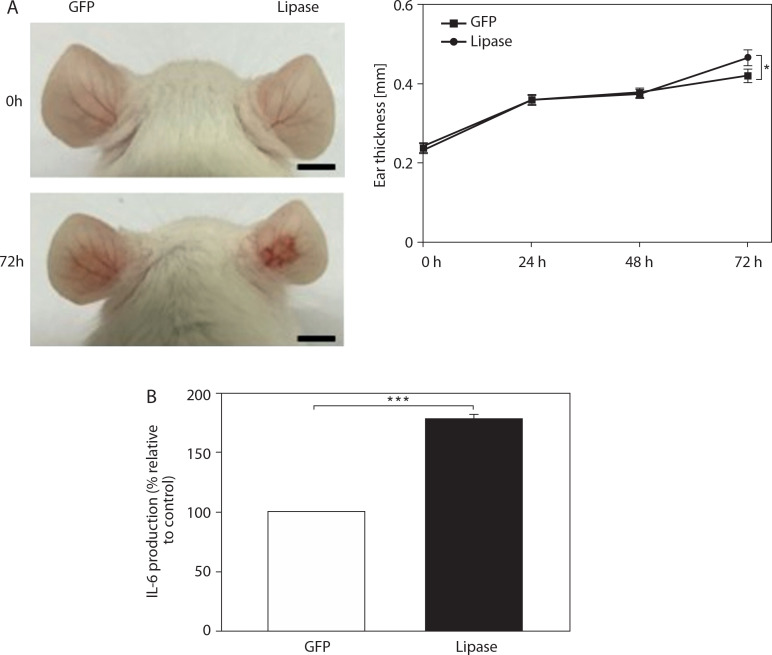
The immunogenicity of *Cutibacterium acnes* lipase in mice. The ears of mice were intradermally injected with 50 μg lipase or green fluorescent protein (GFP) and observed for 72 h. (**A**) Ear morphology and changes in ear thickness (mm) are shown. Scale bar = 1 mm. (**B**) The production of pro-inflammatory interleukin-6 (IL-6) was quantified by enzyme-linked immunosorbent assay (ELISA). Data are expressed as mean ± standard deviation (*p < 0. 05; **p < 0.01; ***p < 0.001 vs. control, *n* = 3)

**Figure 5:**
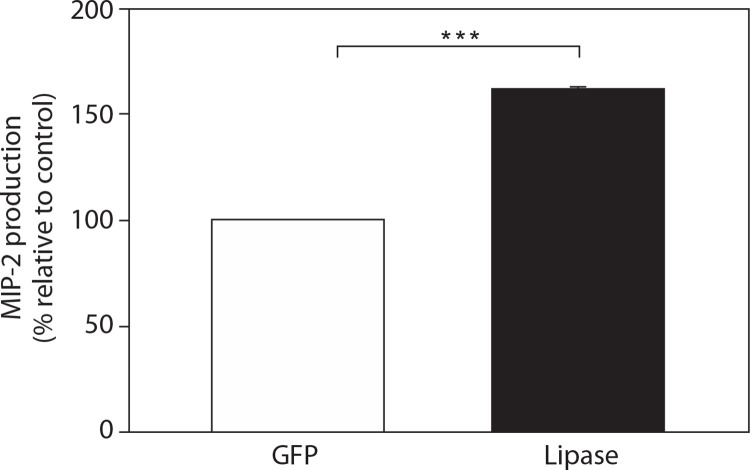
The production of macrophage inflammatory protein-2 (MIP-2) and the immunogenicity of *Cutibacterium acnes* lipase in mice. The ears of mice were intradermally injected with 50 μg lipase or green fluorescent protein (GFP) and observed for 72 h. The production of MIP-2 was quantified by enzyme-linked immunosorbent assay (ELISA). Data are expressed as mean ± standard deviation (*p < 0.05; **p < 0.01; ***p < 0.001 vs. control, *n* = 3)

To investigate lipase as an immunogenic antigen and confirm its virulence, mice were intradermally injected with 50 μg of lipase or GFP into the right or left ears, respectively, and ear thickness was measured after 24, 48, and 72 h post-injection (Figure 4A). The ears of mice injected with lipase exhibited a relatively high level of inflammation, characterized by visible swelling and increased ear thickness, compared to those of mice injected only with GFP. These findings suggest that lipase plays a key role in virulence activity, as demonstrated by the localized inflammation in mice following lipase injection. Mice injected with lipase also exhibited significantly elevated levels of pro-inflammatory IL-6 (Figure 4B) and MIP-2 (Figure 5), suggesting that lipase possesses high virulence potential and induces excessive infection. Previous studies have shown that pro-inflammatory IL-6 and MIP-2 expression mediates acute-phase responses and drives innate immune activation (Josse et al. 2020; Flori et al. 2023; Negari et al. 2025). These findings indicate that *C. acnes* lipase exhibits immunogenic properties in mice vaccinated with the recombinant *C. acnes* lipase. This aligns with other research reporting that *C. acnes* lipase is immunogenic and contributes to the inflammatory processes involved in acne vulgaris (Mayslich et al. 2021).

### Protective immunity by anti-lipase/C. acnes in mice

**Figure 6:**
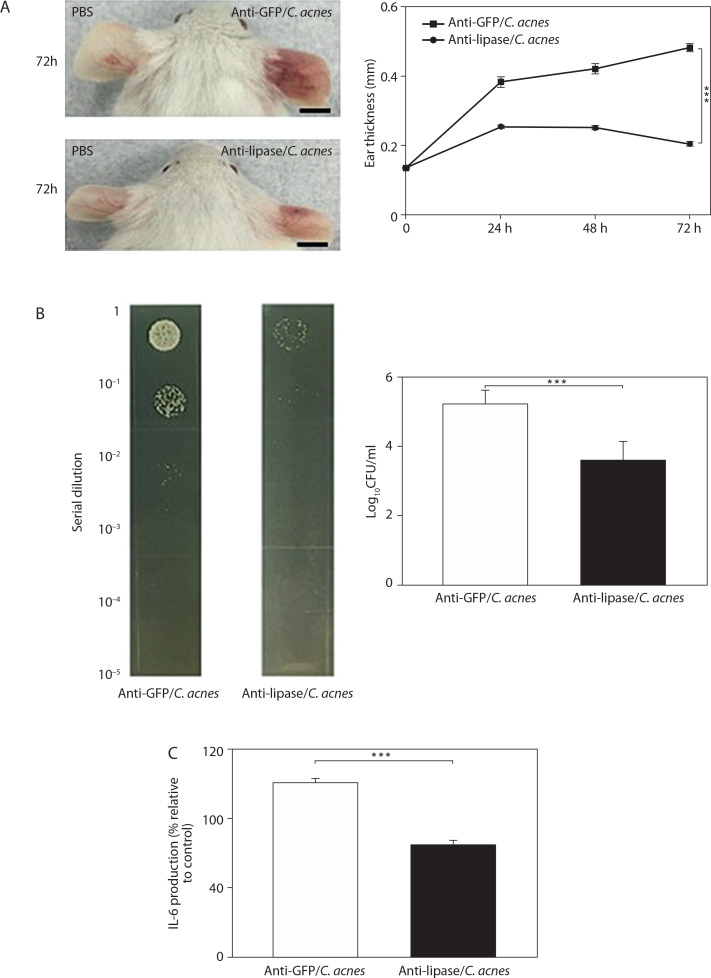
Immune protection by anti-lipase/*Cutibacterium acnes* (*C. acnes)*. *C. acnes* (10^7^ CFU) or phosphate-buffered saline (PBS) was injected into the right or left ears, respectively, of mice vaccinated with lipase or green fluorescent protein (GFP) and incubated for 72 h. (**A**) Ear morphology and changes in ear thickness (mm) are shown. Scale bar = 1 mm. (**B**) The CFUs (log_10_CFU/ml) were enumerated by plating serially diluted (1:10^0^–1:10^5^) ear homogenates on agar plates. (**C**) The production of pro-inflammatory interleukin-6 (IL-6) was quantified by enzyme-linked immunosorbent assay (ELISA). Data are expressed as mean ± standard deviation (*p < 0.05; **p < 0.01; ***p < 0.001 vs. control, *n* = 3)

To evaluate the protective immune efficacy and assess the effectiveness of lipase vaccination in reducing *C. acnes*-induced inflammation, 10 μl of *C. acnes* (10^7^ CFU) was administered intradermally into the right ears of mice vaccinated with anti-lipase or GFP, while the left ears were injected with 10 μl of PBS to serve as controls. Inflammatory responses were monitored at 24, 48, and 72 h post-injection. As shown in Figure 6A, mice injected with anti-lipase/*C. acnes* showed significantly reduced ear redness and thickness, compared to mice injected with anti-GFP/*C. acnes*. Furthermore, the bacterial load, determined by CFU/ml counts from serial dilutions (1 : 10^0^–1 : 10^5^) of ear homogenates, was notably lower in the anti-lipase/*C. acnes* group (Figure 6B). Given that acne vulgaris is characterized as an inflammatory skin condition (Ahle et al. 2023; Dréno et al. 2018; Kim et al. 2020), we further investigated whether anti-lipase/*C. acnes* treatment influenced the production of pro-inflammatory IL-6 in mice (Figure 6C). As expected, anti-lipase/*C. acnes* treatment markedly decreased the production of pro-inflammatory IL-6, suggesting that vaccination with *C. acnes* lipase elicited protective immunity against *C. acnes* by neutralizing its secretory virulence activity.

### Passive immunization with neutralization antibodies to C. acnes lipase

**Figure 7:**
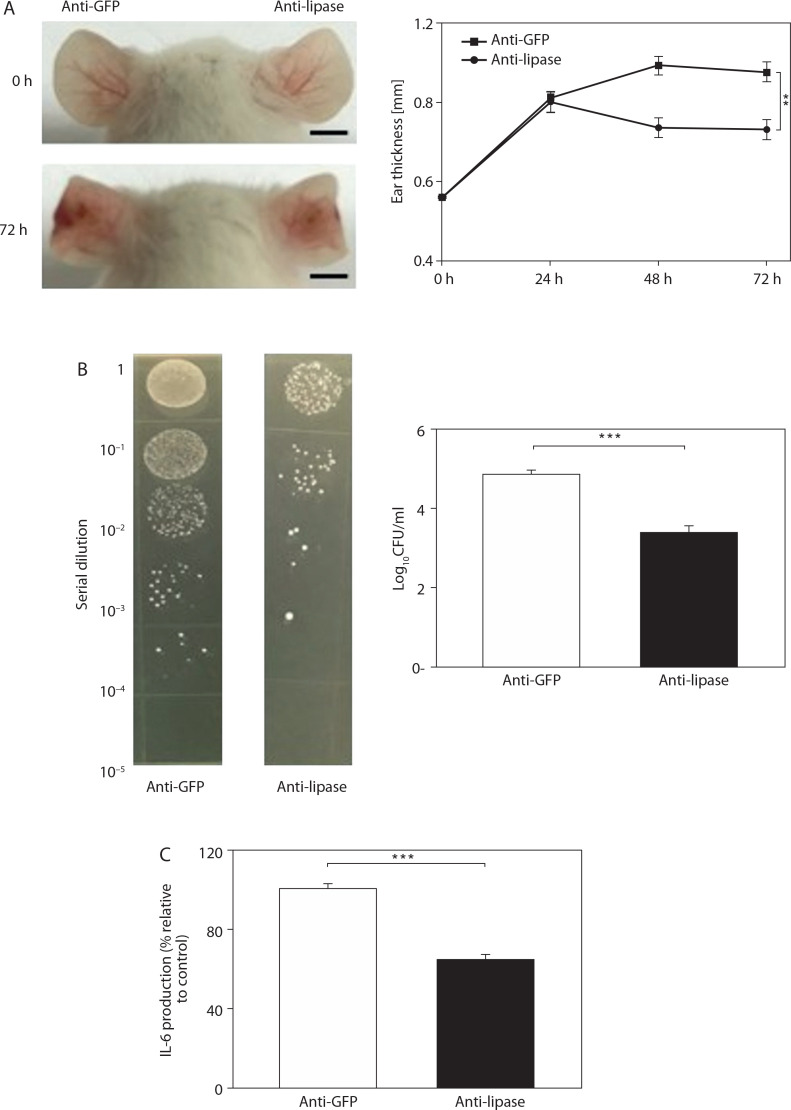
Neutralization with anti-lipase serum decreases *Cutibacterium acnes* (*C. acnes*)-induced bacterial growth and the production of pro-inflammatory interleukin-6 (IL-6) in mice. *C. acnes* was pre-treated with 5% (v/v) anti-lipase or green fluorescent protein (GFP) serum at 37°C for 1 h. Pre-treated *C. acnes* (10^7^ CFU) was then applied onto the ears of the Institute of Cancer Research mice and incubated for 72 h. (**A**) Ear morphology and changes in ear thickness (mm) are shown. Scale bar = 1 mm. (**B**) The CFUs (log_10_CFU/ml) were enumerated by plating serially diluted (1:10^0^–1:10^5^) ear homogenates on agar plates. (**C**) The production of pro-inflammatory IL-6 was quantified by enzyme-linked immunosorbent assay (ELISA). Data are expressed as mean ± standard deviation (*p < 0.05; **p < 0.01; ***p < 0.001 vs. control, *n* = 3)

**Figure 8:**
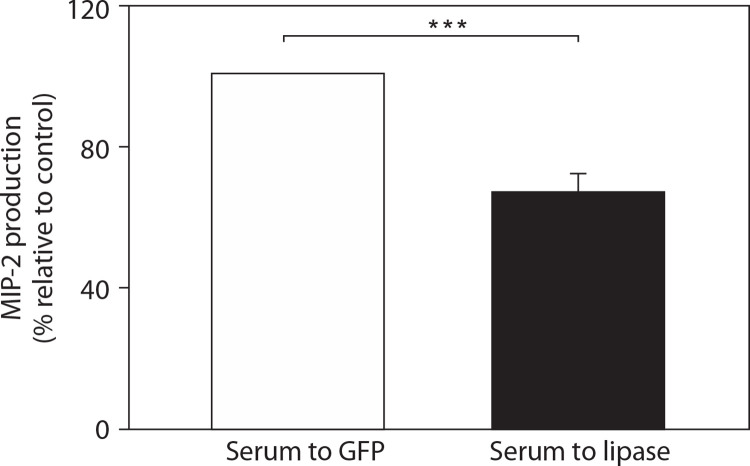
Neutralization with anti-lipase serum decreases *Cuti bacterium acnes* (*C. acnes*)-induced the production of macrophage inflammatory protein-2 (MIP-2) in mice. *C. acnes* was pre-treated with 5% (v/v) anti-lipase or green fluorescent protein (GFP) serum at 37°C for 1 h. Pre-treated *C. acnes* (10^7^ CFU) was then applied onto the ears of the Institute of Cancer Research mice and incubated for 72 h. The production of MIP-2 was quantified by enzyme-linked immunosorbent assay (ELISA). Data are expressed as mean ± standard deviation (*p < 0.05; **p < 0.01; ***p < 0.001 vs. control, *n* = 3)

**Figure 9:**
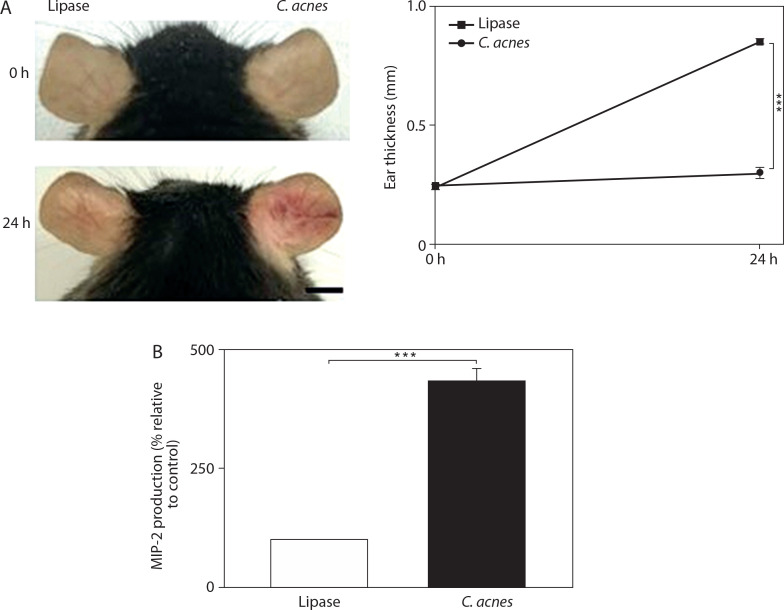
The reduction of macrophage inflammatory protein-2 (MIP-2) in the pro-inflammatory interleukin-6 (IL-6) knockout mouse. The ears of the pro-inflammatory IL-6 knockout mice were intradermally injected with lipase as a control or *Cutibacterium acnes* (10^7^ CFU) and observed for 24 h. (**A**) Ear morphology and changes in ear thickness (mm) are shown. Scale bar = 1 mm. (**B**) The production of MIP-2 was quantified by enzyme-linked immunosorbent assay (ELISA). Data are expressed as mean ± standard deviation (*p < 0.05; **p < 0.01; ***p < 0.001 vs. control, *n* = 3)

We analyzed whether passive immunization with neutralization antibodies to *C. acnes* lipase can decrease *C. acnes-*induced inflammation. To achieve this, anti-lipase or anti-GFP serum was heated at 58^o^C for 30 min to inactivate other protein complements in the serum. The complements were pre-treated with 5% (v/v) inactivated anti-lipase serum or GFP in the PBS solution at 37°C for 1 h. Subsequently, the serum was added to *C. acnes* (10^7^ CFU) in media and incubated at 37^o^C for 1 h. The resulting mixture was intradermally injected into the right or left ears of mice, and inflammation was evaluated at 24, 48, and 72 h post-injection. Results showed that the ears injected with anti-lipase serum neutralized *C. acnes* and exhibited less inflammation in terms of both redness and thickness, compared with ears treated with anti-GFP serum (Figure 7A). Moreover, the anti-lipase serum significantly reduced bacterial colony counts (CFU/ml), as determined from serially diluted samples (1 : 10^0^–1 : 10^5^) of ear tissue homogenates, compared to the anti-GFP serum (Figure 7B). We further analyzed the levels of pro-inflammatory cytokines in the lipase-injected ears and observed decreased levels of IL-6 (Figure 7C) and MIP-2 (Figure 8). In addition, we evaluated the reduction of MIP-2 in the IL-6 knockout mouse (Figure 9) to determine whether this pro-inflammatory cytokine is influenced by *C. acnes* and lipase induction, thereby indicating whether MIP-2 may also serve as a marker of *C. acnes*-induced inflammation. This hypothesis is supported by the results shown in Figure 9, where IL-6 knockout mice injected with *C. acnes* or lipase exhibited visible ear redness and increased ear thickness (Figure 9A). According to previous studies, the levels of MIP-2 contributes significantly to the activation of innate immunity, and the elevated levels of MIP-2 cytokines induced by lipase in acne vulgaris may result in severe inflammation and the production of other proinflammatory cytokines (Wang et al. 2018; Parvin et al. 2020; Marito et al. 2020; Kao et al. 2022). Therefore, we analyzed the expression of MIP-2 to confirm its key role in *C. acnes*-induced inflammation. As shown in Figure 9B, the inflammatory response in mice injected with *C. acnes* was higher than that in mice injected with lipase alone, as indicated by elevated MIP-2 production. These findings suggest that passive immunization with neutralizing antibodies against lipase efficiently inhibits the secretion of the pro-inflammatory cytokines IL-6 and MIP-2, while simultaneously reducing bacterial colonization. Additionally, a marked increase in MIP-2 production was observed in IL-6 knockout mice, highlighting a compensatory inflammatory response in the absence of IL-6.

**Figure 10:**
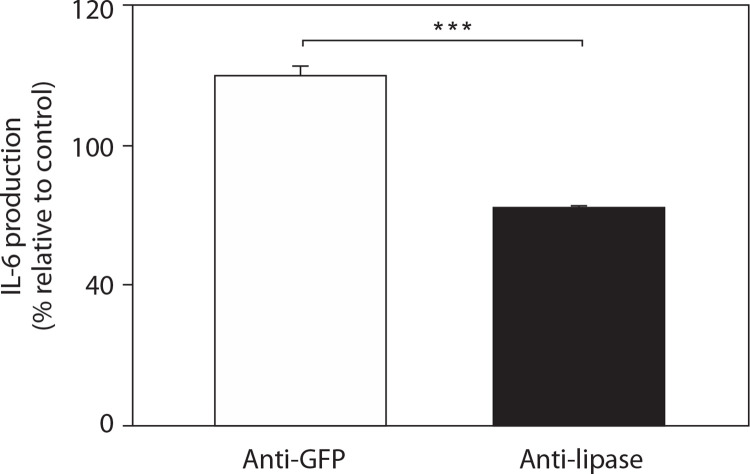
Neutralization with anti-lipase serum decreases *Cutibacterium acnes* (*C. acnes*)-induced the production of pro-inflammatory interleukin-6 (IL-6) in sebocytes. *C. acnes* was pre-treated with 5% (v/v) anti-lipase or green fluorescent protein (GFP) serum at 37°C for 1 h. Pre-treated *C. acnes* (10^7^ CFU) was then co-cultured with sebocytes (8 × 10^5^) for 12 h. The production of pro-inflammatory IL-6 was quantified by enzyme-linked immunosorbent assay (ELISA). Data are expressed as mean ± standard deviation (*p < 0.05; **p < 0.01; ***p < 0.001 vs. control, *n* = 3)

Thus, we also evaluated the ability of the anti-lipase serum to inhibit *C. acnes*-induced IL-6 expression in sebocytes. Cells exposed to *C. acnes* pre-treated with either anti-lipase or anti-GFP serum secreted IL-6, with notable differences observed between the two conditions (Figure 10). These results demonstrated that passive immunization with neutralizing anti-lipase serum effectively decreases *C. acnes*-induced IL-6 production in sebocytes. This finding aligns with previous studies indicating that sebocytes located in the sebaceous glands serve as the primary targets for *C. acnes* in individuals affected by acne vulgaris (Schneider and Zouboulis 2018).

## Discussion

In this study, we explored the contribution of *C. acnes* in acne pathogenesis, which appears to be associated with its production of enzymes that degrade host molecules, including lipases, proteases, hyaluronidases, and acid phosphatases (Brown 1998; Jappe 2003). In addition, *C. acnes* possesses a distinctive cell wall and membrane structure containing phosphatidylinositol, triacylglycerol, and other lipid components (Jeon et al. 2018). Among these enzymes, *C. acnes* lipase (GehA, glycerolester hydrolase A) is the primary enzyme responsible for hydrolyzing sebum triglycerides into glycerol and free fatty acids (Jappe 2003). Our GC–MS analysis revealed that *C. acnes* lipase can degrade the lipids in sebocytes into various fatty acids. As a key bacterium associated with acne, *C. acnes* obtains energy through the breakdown of sebum lipids via lipase activity (Kim et al. 2020). Two compounds were found to be significantly increased (*p* < 0.05) after lipase treatment and were identified as palmitoleic acid and palmitic acid. Structural studies of type II *C. acnes* lipase have shown that the active site of this enzyme is concealed beneath a hydrophobic lid region, and the surrounding lipid environment influences its catalytic activity (Kim et al. 2020).

According to previous studies, palmitic acid can suppress hydrogen peroxide production by neutrophils. By inducing oxidative stress and the disruption of epidermal barrier function, this process facilitates the penetration of pro-inflammatory mediators through hair follicles into the dermis, thereby exacerbating acne-related inflammation (Lovászi et al. 2017; Nakamizo et al. 2021; Burkhart 2024; Akamatsu et al. 2008; Brown 1998). Fatty acids, such as unsaturated palmitoleic acid and saturated palmitic acid, are also among the most potent antimicrobial components in human sebum (Simonart 2013; Lovászi et al. 2017; Choi et al. 2019). Treatment of sebocytes with these fatty acids, particularly palmitic acid, can result in significantly increased pro-inflammatory IL-6 expression. Lipase generated by *C. acnes* is therefore considered an important determinant of bacterial pathogenicity, working together with other virulence factors (Kim et al. 2020). Free fatty acids, such as oleic acid and palmitic acid, are associated with acne severity. Palmitate is the predominant saturated fatty acid bound in neutral lipids, mainly triglycerides, which constitute 40–60% of sebum by weight and can induce lipid accumulation and inflammatory responses in sebocytes (Tabri 2019; Choi et al. 2019; Nakase et al. 2022; Flori et al. 2023; Kovács et al. 2023). We also observed relatively high cholesterol levels in both GFP- and lipase-treated samples from sebocytes. Previous studies have reported that sebocytes contain substantial amounts of cholesterol, triglycerides, and wax esters, and that cholesterol plays an important role in maintaining membrane integrity, epidermal barrier formation, and sebum secretion (Knox and O’Boyle 2021; Briganti et al. 2021; Flori et al. 2023; Yin et al. 2024).

Previous studies have used ICR mice as a model for inflammatory responses to assess the efficacy of inactivated *C. acnes*-based vaccines (Nakatsuji et al. 2008; Liu et al. 2011). An outgrowth of *C. acnes*, along with the release of virulence factors, enzymes, and pattern recognition ligands, stimulates cutaneous inflammation and contributes to acne lesion formation (Hazarika 2021; Singh et al. 2022; Ahle et al. 2023). Among these mediators, the enzymatic activity of *C. acnes* lipase has been identified as a key contributor to acne pathogenesis (Dréno et al. 2018; Josse et al. 2020; Singh et al. 2022). In the present study, ICR mice were subcutaneously vaccinated with lipase or GFP into the dorsal skin three times at 2-week intervals. ELISA results showed a significantly elevated antibody titer to lipase following vaccination. Previous studies have also reported increased serum antibody titers against *C. acnes* in patients with inflammatory acne compared with healthy individuals (Mayslich et al. 2021; Huang et al. 2021). Immunochromatographic test strip analysis further demonstrated that lipase or *C. acnes* lysates were recognized by antibodies in both anti-lipase serum and *C. acnes*, indicating that vaccination with lipase provoked antibodies that can cross-react with *C. acnes*. These results demonstrate the immunogenic potential of lipase to induce antibody production and its crossreactivity with serum lipase, suggesting its contribution to acne vulgaris. Therefore, targeting *C. acnes* lipase through vaccination may represent a potential strategy for preventing early-stage *C. acnes*-mediated infection. The finding that immunization with lipase markedly elevated antibody titers confirms its strong immunogenic potential (O’Neill and Gallo 2018; Lee et al. 2019; Zhang et al. 2025).

Lipase also exhibited immunogenic properties when injected intradermally into mouse ears, as indicated by increased ear thickness in mice at 24, 48, and 72 h post-injection. This consistent swelling indicated that lipase alone can trigger a localized inflammatory response in vivo (Josse et al. 2020; Kim et al. 2020). To further investigate this effect, the pro-inflammatory IL-6 levels in lipase-injected ears were measured by ELISA and found to be significantly elevated. In addition, the level of MIP-2 was increased, indicating a robust immune response. These findings highlight the pro-inflammatory role of lipase in initiating immune activation and support its contribution as a key virulence factor in *C. acnes*-induced skin inflammation, further confirming its immunogenic potential (Mayslich et al. 2021; Korn and Hiltensperger 2021).

Lipase also plays a crucial role in *C. acnes*-induced inflammation, as demonstrated by the attenuation of inflammatory responses in mice administered with anti-lipase/*C. acnes*. A reduction in bacterial load within mouse ears further suggests that lipase vaccination inhibits bacterial proliferation and promotes clearance via innate immune defenses. The pro-inflammatory cytokine IL-6, which plays a key role in innate immunity, also showed reduced expression. This demonstrates successful induction of antibody production in lipasevaccinated mice, reflecting the activation of adaptive immune responses (Tanaka et al. 2014; Kim et al. 2020; Kang et al. 2020).

Passive immunization with neutralizing antibodies to lipase significantly reduced *C. acnes*-induced inflammation in mice, indicating the potential of targeted antibody therapy to modulate skin immune responses. In this study, mice were intradermally injected with *C. acnes* (10^7^ CFU), combined with either anti-lipase or anti-GFP serum, in the ears. Inflammation was assessed by measuring ear thickness at 24, 48, and 72 h post-injection. A marked reduction in swelling was observed in the ears treated with anti-lipase serum, indicating that passive transfer of lipase-specific antibodies effectively attenuated local inflammation (Mayslich et al. 2022). To further evaluate bacterial persistence, ear tissue homogenates were serially diluted (1 : 10^0^–1 : 10^5^) and plated to enumerate CFUs. The results showed a modest but consistent reduction in bacterial load in the anti-lipase serum group, supporting the hypothesis that antibody-mediated neutralization of lipase can impair bacterial colonization or viability (Josse et al. 2020). In addition, pro-inflammatory IL-6 and MIP-2 levels were analyzed to evaluate the inflammatory environment at the injection site. Notably, the levels of IL-6, which mediates acute-phase responses and innate immune activation (Zayabaatar et al. 2023; Zhao et al. 2025), were significantly lower in the ears treated with anti-lipase serum. The levels of MIP-2 are also associated with acne pathogenesis and can contribute to severe inflammation and the production of other pro-inflammatory cytokines (Wang et al. 2018; Parvin et al. 2020; Marito et al. 2020; Kao et al. 2022). The reduction in the levels of pro-inflammatory IL-6 and MIP-2 further supports the immunomodulatory effect of lipase-specific antibodies and indicates the suppression of local inflammatory responses (Choi et al. 2019; Wang et al. 2023).

Given the role of the pro-inflammatory cytokines IL-6 and MIP-2 in acne inflammation, we further evaluated MIP-2 levels in IL-6 knockout mice. Mice injected with *C. acnes* exhibited more pronounced inflammatory symptoms, including increased ear thickness and redness, compared with those injected with lipase alone. This was accompanied by increased MIP-2 production, highlighting that this cytokine contributes to the inflammatory response induced by *C. acnes*. These findings are consistent with previous reports identifying MIP-2 as a key component of innate immune defense during the early stages of inflammation (Wang et al. 2018; Parvin et al. 2020; Kao et al. 2022). Higher MIP-2 levels in the *C. acnes* group suggest that this cytokine may not only act as a downstream effector but also amplify local inflammatory responses. This finding highlights the therapeutic potential of targeting lipase antigens isolated from *C. acnes* to control inflammation and microbial imbalance in acne vulgaris. *C. acnes* also stimulates the externalization of neutrophil lysosomal hydrolytic enzymes (Zhang et al. 2024; Yu et al. 2024). Pro-inflammatory cytokines, including IL-6, are critically involved in acne pathogenesis (Jin et al. 2023; Huang et al. 2024; Xu et al. 2024). Crucially, the interaction of *C. acnes* with the inflammatory system can be mitigated with antigen-specific antibodies, suggesting an important role for antibodies against *C. acnes* lipase and pro-inflammatory cytokines in attenuating inflammatory acne.

In addition to passive immunization with neutralizing antibodies to lipase, experiments in sebocytes showed significantly reduced *C. acnes*-induced IL-6 production. Anti-lipase serum added to sebocytes co-cultured with *C. acnes* significantly reduced inflammation, as indicated by decreased pro-inflammatory IL-6 expression. These findings suggest that *C. acnes* lipase may regulate inflammatory responses by influencing local immune reactions or bacterial virulence mechanisms. This aligns with previous studies reporting decreased pro-inflammatory IL-6 expression following lipase neutralization in sebocytes (Flori et al. 2023). Overall, these results support the therapeutic potential of targeting *C. acnes* lipase through immunization strategies and antibody-mediated modulation of sebocyte inflammatory responses.

## Conclusions

This study highlights that secretory lipase derived from *C. acnes* acts as a key contributor in the pathogenesis of acne vulgaris by participating in lipid metabolism and inducing pro-inflammatory responses in sebocytes. Mass spectrometric analysis confirmed that *C. acnes* lipase degrades lipids in sebocytes into various fatty acids, notably palmitic acid, which significantly upregulates the proinflammatory IL-6 expression in these cells. Vaccination with *C. acnes* lipase elicited high-titer IgG antibodies in mice, leading to a significant reduction in bacterial colonization and the levels of the pro-inflammatory cytokines IL-6 and MIP-2. Furthermore, passive immunization with neutralization antibodies to lipase effectively attenuated the inflammatory response, highlighting the immunomodulatory potential of targeting this virulence factor. Collectively, these findings suggest that vaccination targeting *C. acnes* lipase mitigates *C. acnes*-induced inflammation, offering an antigen-specific strategy for both the prevention and treatment of acne vulgaris.

## Supplementary Material


