# Supplementary material for: Acne vaccine targeting Cutibacterium acnes secretory lipase suppresses the bacteria-induced pro-inflammatory interleukin-6 and macrophage inflammatory protein-2 production
Source: BioTechnologia (Pozn). 2026 Jun 27;107(2):163–80. doi: 10.5114/bta/221530 (PMC13409370; doi:10.5114/bta/221530)
Supplement: Supplementary file 1 [file BTA-107-2-221530-s1.pdf]

Supplementary Table 1. The list of fatty acids detected by gas chromatography–mass spectrometry

No.	Compound	Rt. (min)	Peak area (%, n = 3)		Lipase/ GFP	p-value
			GFP	Lipase		
1.	Methyl oleate	0.8	1.69 ±0.43	1.25 ±0.37	0.73	ns
2.	Oleic acid	1.7	6.89 ±0.83	5.27 ±1.03	0.76	ns
3.	Palmitoleic acid	3.3	9.57 ±1.91	13.18 ±2.37	1.38	*
4.	Palmitic acid	4.2	35.54 ±3.36	43.35 ±4.92	1.22	*
5.	Linolenic acid	4.8	3.86 ±0.75	2.38 ±0.92	0.62	ns
6.	Linoleic acid	5.3	3.77 ±0.69	2.64 ±0.55	0.70	ns
7.	Squalene	5.8	7.76 ±0.77	5.09 ±0.84	0.66	ns
8.	Cholesterol	6.1	22.20 ±4.64	20.61 ±3.58	0.93	ns
9.	1,2-dioleoyl-sn-glycerol	7.1	1.69 ±0.43	1.25 ±0.37	0.74	ns
10.	Cholesterol palmitate	7.7	3.41 ±0.42	2.53 ±0.71	0.74	ns
11.	1,3-dipalmitoyal-2-oleoylglycerol	9.2	1.54 ±0.27	1.11 ±0.19	0.72	ns
12.	1-palmitoyl-2-oleoyl-3-lineoleoyl-rac-glycerol	9.6	1.82 ±0.39	1.17 ±0.42	0.64	ns

The *p*-values of < 0.05 (*) were considered statistically significant.
GFP – green fluorescent protein, ns – not significant, Rt. – retention time
